# Equitable access to COVID-19 diagnostics: factors associated with the uptake of rapid antigen testing in Victoria, Australia, January – February 2022

**DOI:** 10.1186/s12889-023-16838-7

**Published:** 2023-10-11

**Authors:** Erica McCormick, Gabrielle Hales, Frances Ampt, Charles Alpren

**Affiliations:** Western Public Health Unit, Western Health, Sunshine Hospital, 176 Furlong Road, St. Albans, VIC 3021 Australia

**Keywords:** COVID-19, Testing, Diagnostics, Rapid antigen testing, Equity, Engagement, Socio-economic disadvantage, Health inequalities, Health equity

## Abstract

**Background:**

Accessible and accurate diagnostics are critical to control communicable diseases. Uptake of COVID-19 rapid antigen (RA) testing requires physical and financial access to tests, knowledge about usage, motivation, and ability to report results. We sought to understand patterns of and factors associated with RA test uptake in Victoria during a period of high caseload, RA test promotion, and difficulty accessing RA and PCR testing. We hypothesise RA test uptake is indicated by the ratio of cases diagnosed by RA test (probable) to those diagnosed using PCR (confirmed) (p:c).

**Methods:**

Analysing case records, trends in p:c were assessed, between regions, sex, age groups, socio-economic strata and cultural diversity. Logistic regression assessed associations between case classification, and median age, postcode-level socio-economic disadvantage, and proportion overseas-born.

**Results:**

We included 591,789 cases. Mean p:c was lower in socio-economically disadvantaged areas (decile 1 + 2: 0.90 vs. decile 9 + 10: 1.10), and in postcodes where the overseas-born population was above the Victorian average (0.83 vs. 1.05). Conversely, p:c was higher in younger age groups; with no difference between sexes overall. In metropolitan Melbourne, odds of RA test usage increased as socio-economic disadvantage decreased (decile 9 + 10, aOR 1.40, 95%CI 1.37–1.43, vs. decile 1 + 2; p < .001), decreased for cases from areas with a higher overseas-born population (aOR 0.85, 0.83–0.86, p < .001), and with older age.

**Conclusions:**

Reduced uptake of RA tests in Victoria is associated with socio-economic disadvantage, cultural diversity, and older age. Equitable access to COVID-19 diagnostics requires elimination of financial barriers, and greater engagement with culturally diverse and older groups. Inequitable RA test uptake may lead to case under-ascertainment, affecting resource allocation, effective control strategy development, in turn impacting COVID-19 morbidity and mortality, and could indicate relative engagement with response initiatives.

**Supplementary Information:**

The online version contains supplementary material available at 10.1186/s12889-023-16838-7.

## Introduction

Equitable access to accurate diagnostic testing is a vital component of communicable disease control. Until late 2021, diagnosis of SARS-CoV-2 virus in the state of Victoria, Australia, was through nucleic acid amplification (polymerase chain reaction (PCR)), at no cost to the patient, and with financial support offered for those required to isolate [1, 2]. Detections via PCR are notified by laboratories to the Victorian Department of Health and classified as confirmed cases [3]. Rapid antigen (RA) testing has been available in Victoria since late 2021, and self-reported positive RA test results have been included in Victorian case numbers as probable cases since 7th January 2022 [3]. Compared to PCR, RA testing offers an opportunity for an individual to obtain COVID-19 results within 30 min without requiring specific expertise or facilities, at a lower cost to government [4]. Cost is borne by the individual, with an average retail price of $20 per RA test reported in January 2022 [5]. Free RA tests were only available to several groups at the start of 2022, including healthcare staff, patients and those in residential and aged care, for point of care use [6], with reimbursement schemes announced for some eligible disability care recipients [7]. In Victoria, the rollout of RA testing occurred during a surge of COVID-19 infections due to the emergence of the Omicron variant [8–10], which put both Victoria’s PCR testing capacity and availability of RA test kits under significant strain [5].

The pathway from COVID-19 infection to notification as a case involves several steps, and differs between confirmed and probable cases. In Victoria, a probable case requires an infected person to have physical and financial access to an RA test, knowledge about test indication and usage, and the motivation and ability to report a result online in English, or by phone via an interpreter. COVID-19 services and assistance including clinical and financial aid to assist isolation (if eligible) can be granted after self-reporting a positive RA test. In contrast, a confirmed case requires attendance at a testing centre for a free test, where administration and swabs are assisted and results automatically provided and notified to the Department of Health enabling access to assistance. Where detection has occurred by both RA and PCR, the case is recorded as confirmed.

Significant barriers to testing, beyond cost of the test, include knowledge of symptoms, test eligibility, test availability, transport; and financial consequences of a positive result [11]. An Australian study identified an individual’s risk perceptions, the inconvenience of testing, and financial implications, as key reasons people did not seek COVID-19 PCR testing [12]. An evaluation of community-wide RA surveillance in Liverpool, United Kingdom found 20% less testing in the most socially deprived areas, despite tests being free, and lower uptake in Black, Asian, and other minority ethnic groups [13]. Critically, socially disadvantaged and culturally and linguistically diverse (CALD) populations experience higher risk of COVID-19 infection and poorer outcomes both locally and overseas [1, 14, 15].

The Western Public Health Unit (WPHU) provides COVID-19 case, contact and outbreak management to central, west and north-west metropolitan Melbourne (53 postcodes at the time of this analysis), including areas of socio-economic disadvantage. We sought to determine the uptake of RA tests in WPHU compared to Victoria and ascertain the factors associated with RA test uptake across metropolitan Melbourne.

As it is not possible to calculate the total number of RA tests purchased (or otherwise obtained) and used in the population, rather only those where a positive test is recorded, measuring uptake of RA testing presents a unique challenge.

We hypothesise that the uptake of RA tests is indicated by the ratio of probable (RA) to confirmed (PCR) cases, based on several assumptions. First, that most people with access to RA testing would choose this method over PCR due to ease of use and speed of the result. Second, that PCR testing is undertaken for most clinically severe cases (i.e. those presenting to healthcare settings). Third, health-seeking behaviour for severe illness is more consistent across geographical areas and between social strata than for mild illness, given high general access to acute health services in the Australian system. Therefore, a detection by RA test is presumed to be more dependent on socioeconomic conditions than a detection by PCR. Hence, measuring the ratio of probable to confirmed cases (p:c) can demonstrate differences in RA test uptake between cohorts of interest, and inform engagement activities to areas and groups that could benefit from targeted public health action to achieve greater health equity.

## Methods

This cross-sectional study identified all COVID-19 cases diagnosed in Victoria with specimens collected between 7 January and 3 February 2022 recorded in the Department of Health, Victoria COVID-19 Data Model. We extracted de-identified data for all cases recorded as having valid Victorian postcode, including case classification (probable or confirmed), postcode, age, sex, and date of diagnosis. We obtained the Socio-Economic Index for Areas Index of Relative Socio-economic Disadvantage (IRSD) and the proportion of the population born overseas in each case’s postcode of residence from the Australian Bureau of Statistics 2016 census data [16, 17].

We classified cases as metropolitan or regional depending on their residential postcode, assigning metropolitan classification to cases residing within the catchments of the Victorian metropolitan local public health units (LPHUs) and regional to those residing elsewhere [Alpren C, email communication to McCormick E September 2022].

We grouped case data by date, sex, age and IRSD decile, and compared daily probable:confirmed case ratio (p:c) for residents in WPHU to the rest of Victoria for each category.

We selected three broad age categories based on utility for surveillance: children (0–18 years), adults 19 to 69 years, and older adults (70 + years). These age categories represent broad differences in disease risk and clinical severity of COVID-19, with people over 70 most likely to experience hospitalisation or death [1].

We grouped cases by IRSD decile of their postcode of residence, with decile 1 representing the greatest relative socioeconomic disadvantage and decile 10 the least. In Victoria, 65% of the population were born in Australia, with 28% either born overseas and 7% not stated [17]. Based on the proportion overseas born in case’s postcode (POSB), we categorised cases by postcodes where the POSB was less than or equal to the 28%, and POSB greater than 28%, with daily p:c compared between POSB categories.

### Statistical analyses

Multivariate logistic regression assessed the independent effect of IRSD, POSB, age and sex on being classified as a probable case (RA test positive), for cases in metropolitan Melbourne only, due to small case numbers in non-metropolitan areas, and differences in access to testing (proximity to stores, pharmacies, testing sites) between metropolitan and regional areas.

Three metropolitan postcodes did not have population level data, either due to small or no population recorded in census data [16, 17] or due to postcode areas created after the 2016 census [18]; these were excluded from IRSD and POSB analysis. Analyses were conducted using R Studio v4.1.3 [19].

### Ethical approval

The study was approved by the Human Research Ethics Committee of the Western Health Office for Research (QA2022.23).

## Results

Of 595,786 COVID-19 cases notified in Victoria between 7 and 2022 and 3 February 2022 inclusive, 591,789 had a Victorian postcode, with 270,883 probable cases and 320,906 confirmed cases. Metropolitan Melbourne accounted for 479,703 (81%) of cases (Table 1). We found similar age and sex distribution between probable cases and confirmed cases.

**Table 1:** Characteristics of COVID-19 cases – Victoria, 7 January to 3 February 2022

Characteristic	Probable, n = 270,883	± SD	Confirmed, n = 320,906	± SD
Age at diagnosis (years), mean	32.4	18.2	34	18.8
Sex, n (%)		**%**		**%**
Female	141,513	52	164,896	51
Male	128,533	47	154,042	48
Not stated	223	< 0.1	493	0.2
Other	614	0.2	1,475	0.5
Region ^a,b,^ n (%)				
Regional Victoria	52,830	20	58,978	18
Metropolitan Victoria	218,022	80	261,681	82

^a^ Regional borders based on local public health unit’s postcode allocation. Regional Victoria represents combined totals from the 6 regional public health units, metropolitan Victoria combined total from 3 metropolitan Melbourne public health units

^b^ 247 Confirmed cases and 31 probable cases not allocated by region due to postcodes listed as post office boxes

**Fig. 1:**
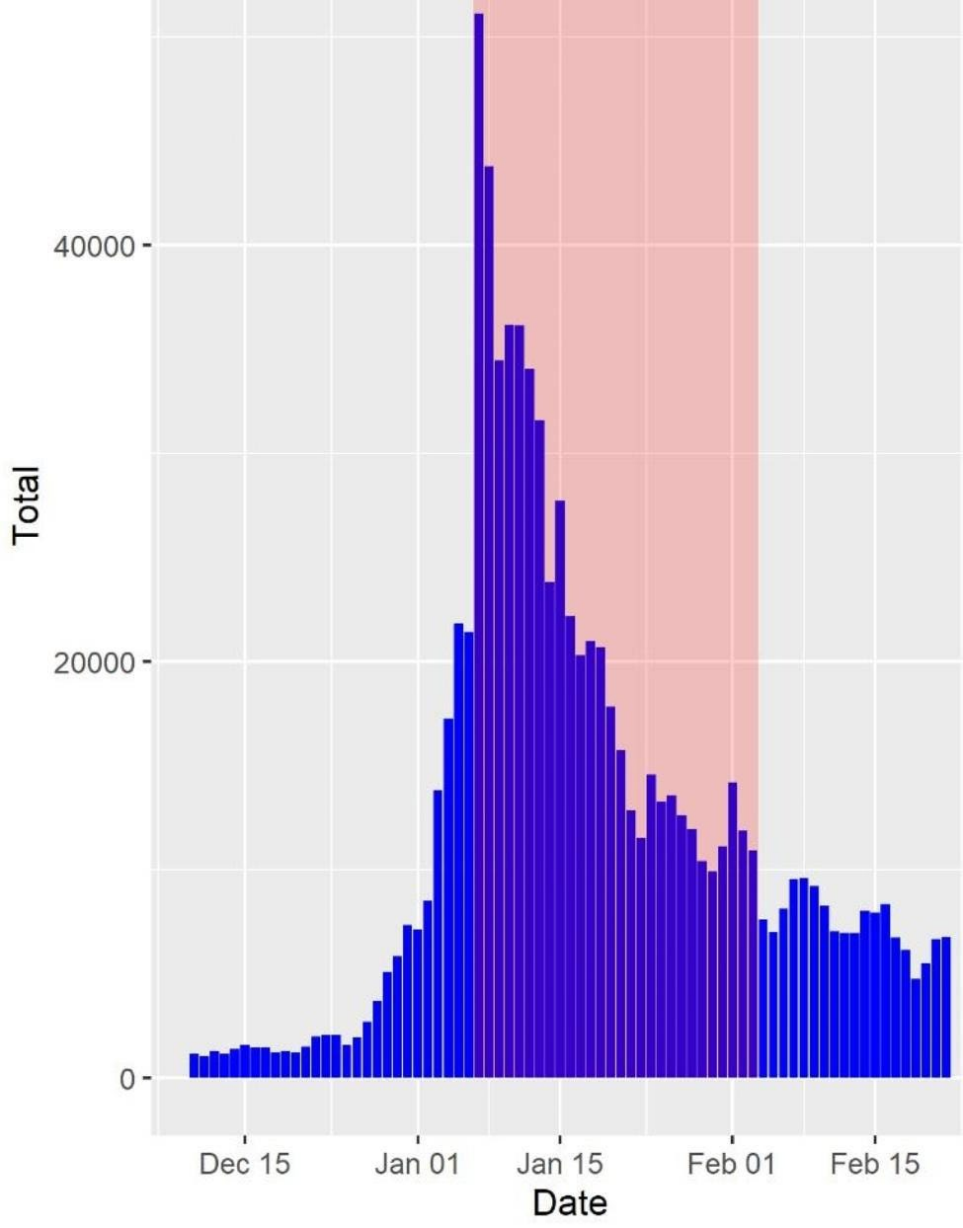
Epidemiological curve of COVID-19 cases by diagnosis date – Victoria, December 2021 to February 2022. The study period is represented by the dates included within the overlaying red area

The study period occurred during a time of high COVID-19 caseload with a peak in daily case incidence on 7 January (50,834), the first day of the study period (Fig. 1).

### Probable to confirmed ratio

**Fig. 2:**
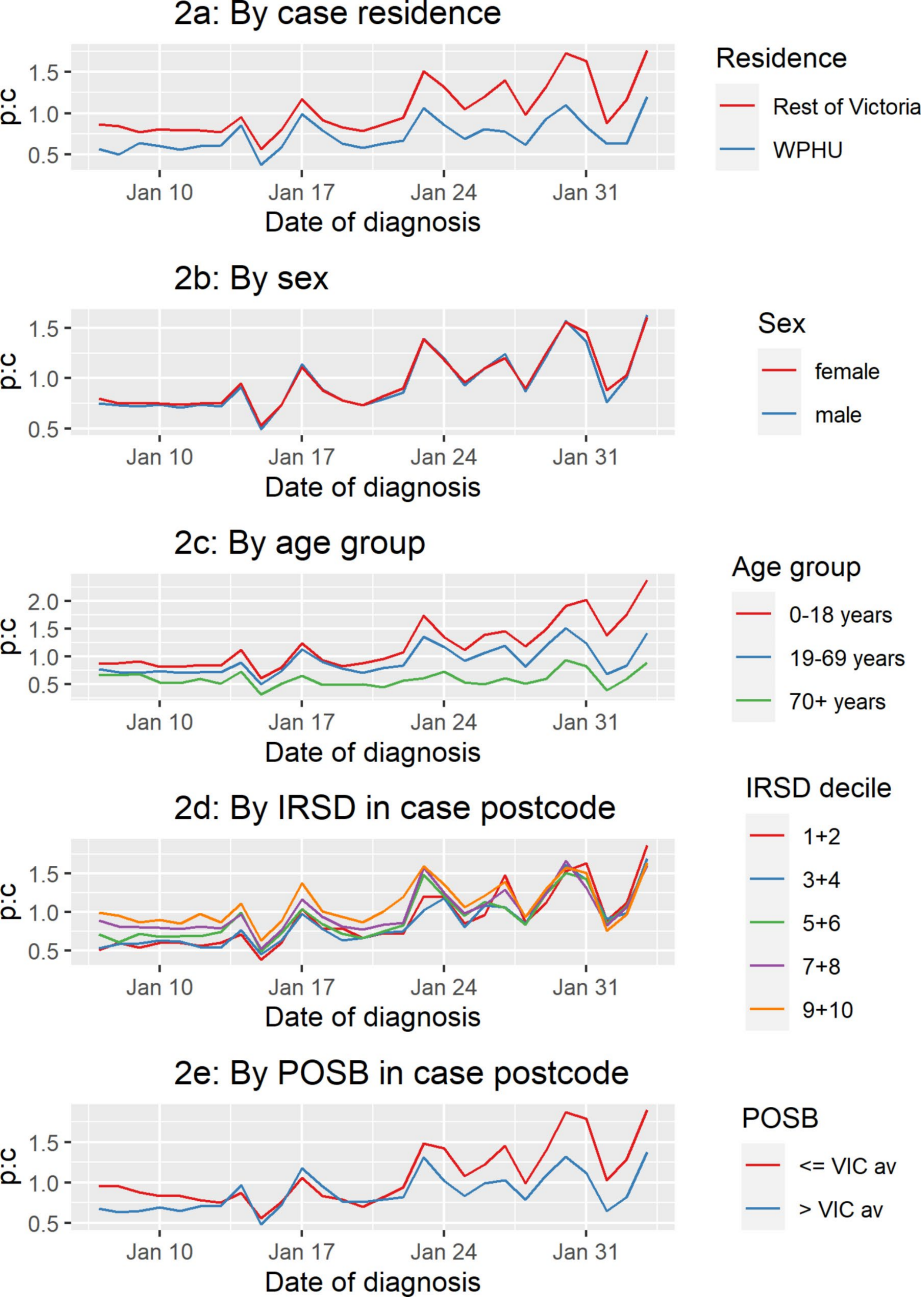
**a-e**: Daily probable to confirmed COVID-19 case ratio – by variables of interest - Victoria

P:c increased gradually in the WPHU catchment and the rest of Victoria, however remained lower in WPHU catchment throughout the study period (Fig. 2a). The mean p:c for the period was 0.73 in WPHU and 1.05 for the rest of Victoria.

### Age and sex

P:c remained similar for males and females throughout the study period (Fig. 2b*).* We saw a greater overall p:c and increase in p:c over time, in younger compared to older age groups (Fig. 2c). The mean p:c for the study period for ages 0–18 years was 1.20 with an increase of 174% over time, compared to a mean p:c of 0.60 and 33% increase over time for those aged 70+.

### IRSD decile

Victorian postcodes with a lower IRSD decile (indicating greater relative socioeconomic disadvantage) had a lower p:c compared to areas with higher IRSD, with relative convergence of the cohorts by the end of the study period (Fig. 2d). IRSD 1 + 2 areas had the lowest mean p:c for period: 0.90; with a 207% increase over the study period; IRSD 9 + 10 had highest mean p:c for period: 1.10; and an increase of 66%.

### Population born overseas

Areas with higher POSB than Victorian average (28%) had a lower p:c throughout most of the period compared to areas with less than or equal to the Victorian average of overseas-born residents (Fig. 2e). Similarly, areas with higher POSB had lower mean p:c for the period; 0.88, with a 103% increase over study period, compared to 1.08 in postcodes with less than or equal to the Victorian average of POSB, and a 100% increase over the study period.

### Regression analyses

**Fig. 3:**
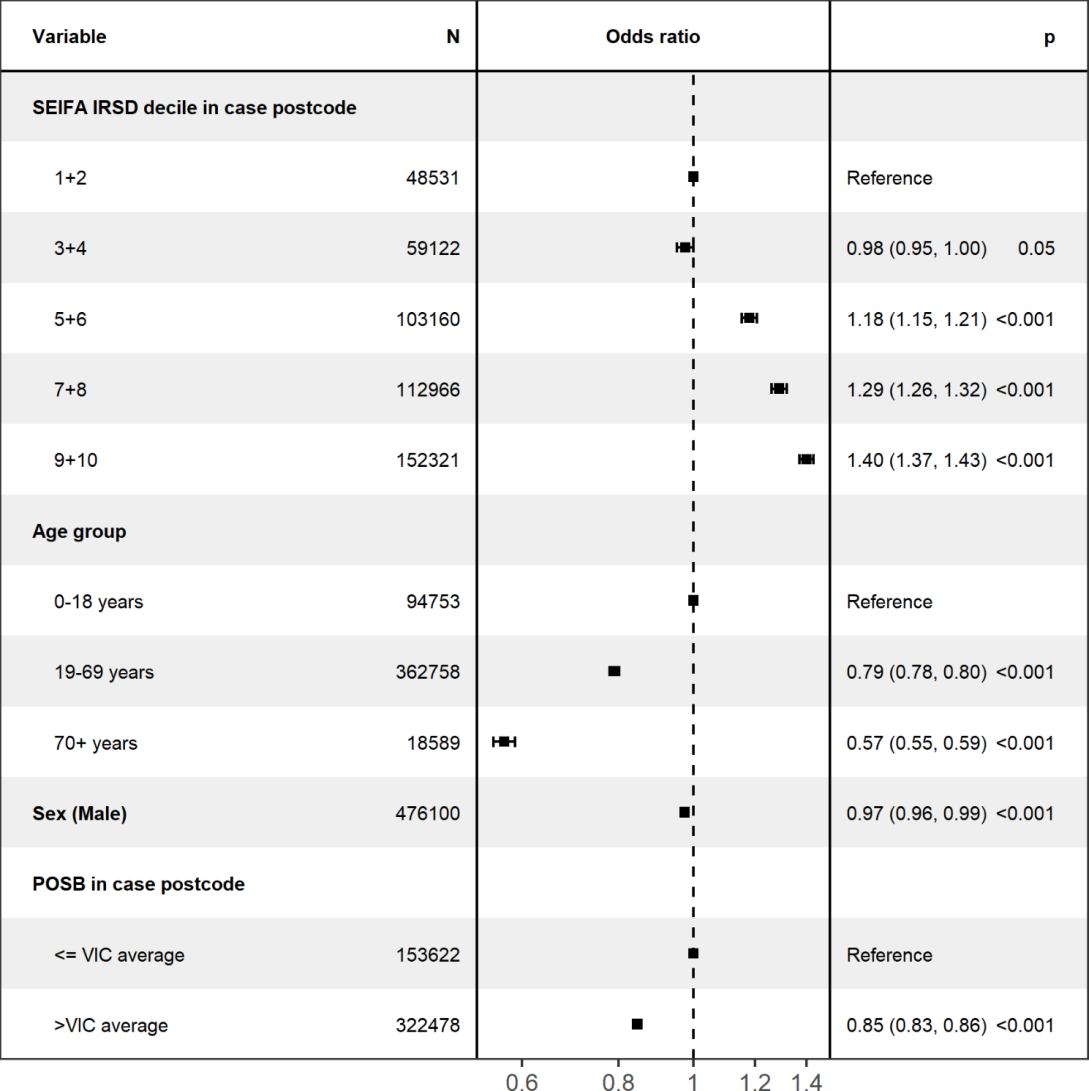
Factors associated with a COVID-19 rapid antigen test on multivariable logistic regression analyses – metropolitan Melbourne **Note**: Estimates represent adjusted odds ratios

Predictors of case classification (probable compared to confirmed) on multivariate logistic regression analysis are presented in Fig. 3. Older age was associated with lower odds of being a probable case. Cases aged 70 years and above had 0.57 times the odds, and ages 19–69 years had 0.79 times the odds of being a probable case, compared to those aged 0–18 years. Living in a postcode area with higher IRSD decile was associated with increased odds of being a probable case (IRSD 9 + 10 aOR 1.40, 95%CI 1.37–1.43). Cases from postcodes where the POSB was greater than the Victorian average, had lower odds (aOR 0.85; 95% CI 0.83–0.86) of being a probable case compared to areas with less than the Victorian average. Males had slightly lower odds of being a probable case compared to females (aOR 0.97, 95% CI 0.96–0.99). All predictor variables had a significant association with the outcome (see Fig. 3). Model fit was assessed using *c* statistic (0.55) and le Cessie-van Houwelingen-Copas-Hosmer global goodness of fit (p < .001) suggesting that much of the variability was attributable to unmeasured factors.

## Discussion

This analysis demonstrates clear differences in COVID-19 testing behaviour between different areas in Victoria, Australia, with lower uptake of RA testing in areas of greater socio-economic disadvantage, and with a higher proportion of overseas-born residents. Accordingly, the odds of diagnosis using RA testing increased with increasing socio-economic advantage and decreased with age.

A surge of COVID-19 cases in Victoria in early 2022 due to the highly transmissible Omicron variant placed significant strain on the diagnostic capacity of laboratories, increasing PCR turnaround-times. At the same time, positive results from RA tests were included in case numbers as probable cases, however this was unlikely to have relieved pressure on testing due to RA test supply limitations and high retail prices [5]. The government had distributed 400,000 RA tests supply via state-run COVID-19 testing centres as of 16 January [20], an average of only 44,000 tests a day where daily case numbers were averaging 31,000 during the same period. The number of Victorians unsuccessfully attempting to access COVID-19 testing at this time remains unknown.

The high cost of a commercially sourced RA test in January may have contributed to the lower uptake in postcodes where there is higher relative socio-economic disadvantage. Those most vulnerable to wage insecurity from the casualised nature of their work are more likely to have lower wages [21] and therefore live in areas with greater socioeconomic disadvantage [17]. These groups may be dissuaded from reporting tests due to the financial consequences of isolating [11, 12, 22] despite financial aid being available for those required to isolate, as applicants must meet specific criteria [2]. The clear divergence in RA test uptake between areas based on relative socio-economic disadvantage, at the time of greatest caseload, when the most could be gained from testing fast to stop transmission highlights the need to provide free testing from the outset of a wave of COVID-19 infections for maximum impact. This is especially pertinent for populations most likely to experience poorer outcomes from COVID-19 infection.

The greater uptake of RA tests seen in younger age groups coincides with the commencement of the school year at end of January 2022 and Victorian Government school surveillance testing programs [23]. Increasing availability of RA tests, including the provision of tests as part of the school scheme may explain the congruence in rates of RA test uptake between areas of greater and lesser socio-economic disadvantage by the end of the study period, as households with children attending school gained access to free test kits.

Engagement with CALD groups has been an evolving process, with communications from the government earlier in the pandemic lacking appropriate translation, contextualised messaging, and collaboration with community leaders to ensure effectiveness [24]. The lower uptake of RA tests in postcodes with above average overseas-born population highlights the need for ongoing and improved engagement in collaboration with CALD communities to ensure better communication around testing. Low digital literacy may be a factor affecting RA test uptake, with elderly people and ethnic minorities less likely to use technology to access healthcare [25]. In addition, the Victorian government reporting portal is in English only, therefore less accessible to non-English speakers. These barriers to technology access may result in under-reporting of positive RA results.

Previous research has highlighted disparities in the uptake of RA testing, even when provided free of charge, with lower uptake in culturally diverse populations and in socio-economically disadvantaged areas, for example in the UK [13]. Nevertheless, the provision of free RA test kits in Victoria would eliminate a key barrier. While PCR tests remain available, free of charge, the expectation that an individual who may be experiencing financial pressure or insecure work would be willing to isolate whilst awaiting the result is unreasonable. The commodification of healthcare, such as the pay-for-RA-test approach, has the potential to further widen inequities for vulnerable groups [22] and is in opposition to the UN Sustainable Development Goals for health that aim to achieve universal health coverage and access to quality essential services [26].

Several changes to policy have occurred since the study period. The Australian federal government implemented a scheme from 24 January to 31 July 2022, enabling access to 10 free RA test kits per three-month period – with a maximum of five in any given month - for concession card holders (aged pensioners, low-income earners, seniors, veterans) [27, 28]. Changes to quarantine requirements in Victoria in May allowed close contacts to avoid week-long quarantine provided a RA test is used five times over seven days: [29] the monthly allowance of free RA test kits on the concessional scheme. Community engagement activities by WPHU in conjunction with local councils and health services, provided 45,000 free RA test kits to over 150 CALD groups in the region during February and March 2022 (internal data), with these efforts replicated across other local public health units. The impact on RA test uptake of this and the government’s concessional access scheme are yet to be evaluated and would benefit from further study.

The implications of low RA test uptake on incomplete case ascertainment is crucial to consider: on a program level, under-detection of cases underestimates the true burden of disease in an area, influencing resource allocation and impeding the ability to plan effective control strategies. At the community level, the impact on morbidity and mortality for Victoria’s most socially vulnerable populations as undetected cases continue to transmit in the community remains to be seen. Further research to explore these impacts is critical.

### Limitations

Our study has several limitations. The outcome measure used has not been validated. The measure depends on several assumptions, however is it unlikely that any potential inaccuracy in assumptions would significantly impair the use of this measure as a surveillance tool. Despite this, the probable to confirmed case ratio is a simple measure that uses readily available data to understand where differences between groups occur. We believe it has utility in contexts such as an epidemic where rapid assessment of a public health response is required, and as an initial step in identifying gaps in a care cascade. Further research would be prudent to understand the validity of this measure.

This study is limited by the self-reported nature of RA tests; however, it is not possible to determine the number of positive but unreported RA tests, and we believe comparing p:c between cohorts remains a useful measure to identify groups requiring further public health action. While broad age categorisations enable detection of surveillance signals for those at highest risk of COVID-19 morbidity and mortality, they do not consider other factors that may influence testing or test reporting behaviour, or exposure risk within age groups.

We analysed socio-economic disadvantage and cultural diversity at the population level (by postcode) because socio-economic information is not collected from individuals during notification. This method does not account for the nuances and variability of communities within a postcode area, and may have under- or over-estimated RA test uptake for certain populations, impacting the accuracy at which public health activities can be targeted to populations in need, on the basis of this research. While socio-economic status was considered as a factor in test RA uptake, potential differences in COVID-19 transmission and testing behaviours between employed and unemployed populations was beyond the scope of this research.

Lastly, the cross-sectional nature of the study design cannot confirm causality between the associated factors and the outcome of RA test use; and does not include other variables which may influence RA test use, such as proximity to available tests, type of employment, or differences in COVID-19 transmission between geographical areas. The statistical significance observed in our regression analysis is attributable to the large sample size, and in addition the model exhibited poor fit, suggesting variability may be explained by other factors.

## Conclusion

To conclude, during a period of intense pressure on the laboratory PCR testing system and limited availability of RA test kits, there was a disparity in the uptake of RA testing in western metropolitan Melbourne and areas with greater socio-economic disadvantage, cultural diversity and in older age groups, possibly indicating barriers to RA test access and engagement, despite efforts to reduce inequities through government policies providing financial support and improved community engagement. Groups already disproportionately affected by the COVID-19 pandemic potentially suffered further as a result of poor access to a vital diagnostic tool at a critical point in the COVID-19 response. Public health policies and interventions that support free testing, encourage or reduce the need for reporting and improve access for all in the community will be critical to prevent further health inequities.

### Electronic supplementary material

Below is the link to the electronic supplementary material.

Supplementary Material 1

## Data Availability

The datasets supporting the conclusions of this article are included within its additional files, or are publicly available.
